# Association between higher dietary lycopene intake and reduced depression risk among American adults: evidence from NHANES 2007–2016

**DOI:** 10.3389/fnut.2025.1538396

**Published:** 2025-04-14

**Authors:** Xiaosong Li, Yuru Lan

**Affiliations:** ^1^School of Public Health, Wuhan University, Wuhan, China; ^2^Division of Oncology, Department of Pediatric Surgery, West China Hospital of Sichuan University, Chengdu, China

**Keywords:** dietary lycopene intake, depression, NHANES, diet, lifestyle

## Abstract

**Introduction:**

Although previous researches have suggested that certain dietary nutrients, such as carotenoids, have an effect on depression, epidemiological evidence on the relationship between lycopene and depression remains limited. This study aimed to investigate the association between dietary lycopene intake and depression risk in American adults.

**Methods:**

Data from 18,664 participants in the National Health and Nutrition Examination Survey (NHANES, 2007–2016) were analyzed, with depression defined by a nine-item Patient Health Questionnaire (PHQ-9) score ≥ 10. Dietary lycopene intake was estimated from the mean of two 24-h dietary recalls. Binary logistic regression and restricted cubic spline (RCS) models were employed to assess the relationship.

**Results:**

Depression prevalence was 8.98%, and adjusted analyses indicated that higher dietary lycopene intake was significantly associated with a reduced depression risk compared to the lowest quartile (ORs for the second, third, and fourth quartiles: 0.851 [95% CI, 0.737–0.982], 0.829 [95% CI, 0.716–0.960], and 0.807 [95% CI, 0.695–0.938], respectively). Additionally, a U-shaped relationship was observed, with a reduction in depression risk associated with dietary lycopene intake ranging from 0 to 10,072 μg/d (*P*-non-linear = 0.017).

**Discussion:**

This study suggested that higher dietary lycopene intake may confer a protective effect against depression in American adults.

## 1 Introduction

Depression is a multifactorial mood disorder characterized by persistent emotional disturbances and substantial health impacts (1). In 2008, the World Health Organization identified major depressive disorder as the third leading contributor to the global burden of disease, projecting it to become the leading cause by 2030, highlighting the urgent need for effective prevention and treatment strategies (2, 3). Although existing antidepressant medications have demonstrated some efficacy, their widespread application is constrained by side effects and significant individual variability in treatment response (4, 5). Therefore, exploring novel strategies for the prevention and treatment of depression, particularly through non-pharmacological approaches like dietary interventions, is of significant scientific and clinical importance.

Growing evidence suggest that dietary nutrients and natural products can alleviate depression through various biological mechanisms (6, 7). Lycopene, a potent natural antioxidant, is abundantly present in red fruits and vegetables, including tomatoes, watermelons, and red grapefruits. Its significant anti-inflammatory and antioxidant properties have been extensively documented in studies on various diseases (8–15). Given the roles of oxidative stress and inflammation in the pathophysiology of depression, lycopene’s unique biological properties make it a promising candidate for addressing depression (16–19). The potential role of lycopene in depression remains underexplored, with limited epidemiological evidence and mechanistic studies on its association with depression risk.

By utilizing data from the National Health and Nutrition Examination Survey (NHANES), this study aims to evaluate the association between dietary lycopene intake and depression risk among American adults. Through this research, we aim to offer a scientific basis for dietary interventions in depression and a theoretical foundation for the use of natural products in mental health.

## 2 Materials and methods

### 2.1 Data sources and study population

**Figure 1:**
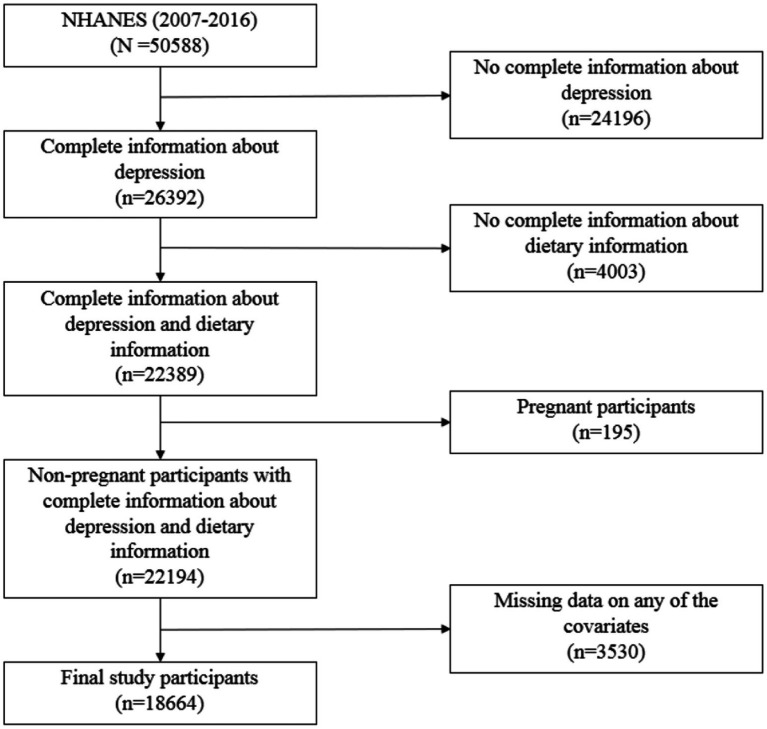
Flowchart of National Health and Nutrition Examination Survey (NHANES) data processing.

This study analyzed data from five consecutive NHANES cycles (2007–2016). After excluding 24,196 participants without complete depression scale data, 4,003 participants with missing dietary data, 195 pregnant participants and 3,530 participants lacking information on any of the covariates, a final sample of 18,664 participants was included (Figure 1). NHANES protocols were approved by the National Center for Health Statistics Ethics Review Board, and all participants provided written informed consent. As this study utilized only publicly available data, no additional ethical approval was necessary.

### 2.2 Dietary lycopene intake

Dietary lycopene intake was determined based on participants’ 24-h dietary recall data, including lycopene content from food, beverages, and water (both tap and bottled). The average of two 24-h dietary recalls was used to calculate individual dietary lycopene intake. Participants were categorized into four groups using the quartile method, with the lowest quartile serving as the reference group.

### 2.3 Depression

Depression was assessed using the nine-item Patient Health Questionnaire (PHQ-9), a validated tool with moderate consistency compared to clinical psychiatric interviews. The PHQ-9 evaluates depressive status through self-reported symptoms (e.g., appetite, self-esteem, sleep quality, and attention) over the preceding 14 days (20). It includes nine questions scored based on participants’ responses, with a total score of ≥10 indicating depression (21).

### 2.4 Covariates

To assess the independent association between dietary lycopene intake and depression risk, we adjusted for potential confounders encompassing sociodemographic, behavioral, and health-related variables. Sociodemographic factors included sex (male, female), age (20–44 years, 45–64 years, ≥65 years), race (non-Hispanic White, non-Hispanic Black, Hispanic American, other racial groups), and education level (less than high school, high school or equivalent, above high school). Family income was categorized based on the poverty income ratio (PIR) into low-income (PIR <1), middle-income (1 ≤ PIR < 4), and high-income (PIR ≥ 4) levels (22). Marital status was classified as married/living with a partner, widowed, divorced/separated, or never married (23).

Behavioral factors included BMI, smoking status, drinking status, physical activity, and dietary quality. BMI was grouped as normal/underweight (BMI ≤ 25), overweight (25 < BMI < 30), and obese (BMI ≥ 30) (24). Smoking status was categorized as never smokers (<100 lifetime cigarettes), current smokers (≥100 lifetime cigarettes), and former smokers (had quit smoking) (25). Drinking status was classified as no (<12 drinks in the past 12 months) or yes (≥12 drinks in the past 12 months). Physical activity data, collected using the Global Physical Activity Questionnaire, was categorized as active (≥600 MET-min/week) or inactive (26, 27). Dietary quality was measured using the Healthy Eating Index-2015 (HEI-2015), calculated from two complete 24-h dietary recalls. Scores ranged from 0 to 100 and were categorized into quartiles (28, 29).

Health status included a history of diabetes, defined as a self-reported physician diagnosis (30). These adjustments ensured robust evaluation of the association between dietary lycopene intake and depression risk.

### 2.5 Statistical analysis

Continuous variables with non-normal distributions are expressed as medians and interquartile ranges, while categorical variables are presented as counts and percentages. Group comparisons of participant characteristics were performed using the Wilcoxon rank-sum test for continuous variables and the *χ*^2^ test for categorical variables. Binary logistic regression models were applied to assess the association between dietary lycopene intake and depression risk. Restricted cubic spline (RCS) model was used to examine potential non-linear relationships, with knots placed at the 5th, 50th, and 95th percentiles of dietary lycopene intake. Statistical analyses were conducted using SPSS software (Version 25.0, SPSS Inc., Chicago, IL, United States), and RCS analyses were performed with the “rms” package in R. All tests were two-tailed, and *p*-values <0.05 were considered statistically significant.

## 3 Results

### 3.1 Basic characteristics of the participants

A total of 18,664 participants aged 20 years or older were recruited for this study, including 9,080 men and 9,584 women. Of the participants, 8.9% were diagnosed with depression. The basic characteristics of all participants are summarized in Table 1. Participants with depression had significantly lower dietary lycopene intake compared to those without depression. Notably, more than half (55.07%) of the participants with depression had dietary lycopene intake below the median intake of the entire participants, while in the group without depression, the proportion exhibited a more evenly distributed dietary lycopene intake. Several covariates showed significant differences between the two groups. Adults with depression were more likely to be female, aged 45–64 years, have a low educational level, have a PIR <1, and be widowed, divorced, or separated. They were also more likely to be obese, current smokers, physically inactive, have lower HEI-2015 scores, have diabetes, and report lower dietary lycopene intake. Further analysis revealed that the median dietary lycopene intake among participants without depression was 2,541 μg/d (interquartile range: 764–6,582 μg/d), whereas the median dietary intake for adults with depression was 2,099 μg/d (interquartile range: 418–5,545 μg/d). The Wilcoxon rank-sum test confirmed a significant difference in dietary lycopene intake between the two groups, suggesting a potential association between dietary lycopene intake and depression risk.

**Table 1:** Characteristics of adults aged 20 years or older according to depression status.

Characteristics	Adults without depression (*n* = 16,988)	Adults with depression (*n* = 1,676)	*p-*value
Gender			<0.001
Male	8,492 (49.99%)	588 (35.08%)	
Female	8,496 (50.01%)	1,088 (64.92%)	
Age group			<0.001
20–44 years	7,074 (41.64%)	677 (40.39%)	
45–64 years	5,767 (33.95%)	722 (43.08%)	
65+ years	4,147 (24.41%)	277 (16.53%)	
Race			0.729
Non-Hispanic White	7,853 (46.23%)	753 (44.93%)	
Non-Hispanic Black	3,477 (20.47%)	351 (20.94%)	
Mexican American	2,407 (14.17%)	237 (14.14%)	
Other races	3,251 (19.14%)	335 (19.99%)	
Education level			<0.001
<High school	3,570 (21.01%)	579 (34.55%)	
High school or equivalent	3,866 (22.76%)	405 (24.16%)	
>High school	9,552 (56.23%)	692 (41.29%)	
Household income			<0.001
PIR <1	3,245 (19.10%)	649 (38.72%)	
1 ≤ PIR <4	9,002 (52.99%)	862 (51.43%)	
PIR ≥ 4	4,741 (27.91%)	165 (9.84%)	
Marital status			<0.001
Married/stable union	10,458 (61.56%)	752 (44.87%)	
Separate/divorced/widowed	3,514 (20.69%)	572 (34.13%)	
Single	3,016 (17.75%)	352 (21.00%)	
BMI status			<0.001
Normal or low weight	4,945 (29.11%)	398 (23.75%)	
Overweight	5,676 (33.41%)	430 (25.66%)	
Obese	6,367 (37.48%)	848 (50.60%)	
Smoking status			<0.001
Never	9,625 (56.66%)	667 (39.80%)	
Former	4,269 (25.13%)	366 (21.84%)	
Current	3,094 (18.21%)	643 (38.37%)	
Drinking status			0.305
No	4,669 (27.48%)	441 (26.31%)	
Yes	12,319 (72.52%)	1,235 (73.69%)	
Physical activity			<0.001
Inactive	6,496 (38.24%)	883 (52.68%)	
Active	10,492 (61.76%)	793 (47.32%)	
HEI-2015			<0.001
<P25	4,083 (24.03%)	583 (34.79%)	
P25 ~ P50	4,224 (24.86%)	442 (26.37%)	
P50 ~ P75	4,287 (25.24%)	379 (22.61%)	
>P75	4,394 (25.87%)	272 (16.23%)	
Diabetes			<0.001
No	14,850 (87.41%)	1,326 (79.12%)	
Yes	2,138 (12.59%)	350 (20.88%)	
Dietary lycopene intake			<0.001
<P25	4,155 (24.46%)	511 (30.49%)	
P25 ~ P50	4,256 (25.05%)	412 (24.58%)	
P50 ~ P75	4,272 (25.15%)	392 (23.39%)	
>P75	4,305 (25.34%)	361 (21.54%)	
Dietary lycopene intake value (μg/d)	2,541 (764, 6,582)	2099 (418, 5,545)	<0.001

PIR refers to the ratio of household income to poverty; BMI refers to the body mass index; HEI refers to the Healthy Eating Index; P25 refers to the 25th percentile/first quartile; P50 refers to the 50th percentile/median; P75 refers to the 75th percentile/third quartile.

### 3.2 Dietary lycopene intake is associated with the risk of depression

Model 1 was adjusted for sociodemographic variables, including sex, age, race, education level, family income, and marital status. Model 2 included additional adjustments for BMI, smoking status, drinking status, physical activity, and the HEI-2015 score. Model 3 further accounted for diabetes. Compared with participants in the lowest quartile of dietary lycopene intake, those in the second, third, and fourth quartiles had odds ratios (ORs) of 0.839 (95% CI, 0.728–0.966), 0.837 (95% CI, 0.724–0.966), and 0.805 (95% CI, 0.695–0.933), respectively, for the risk of depression after adjusting for demographic factors (Table 2). After adjusting for behavioral and health-related confounders, higher dietary lycopene intake remained significantly associated with a reduced risk of depression, with a further increase in dietary lycopene intake linked to an additional decrease in risk.

**Table 2:** Multifactorial analysis of dietary lycopene intake and depression risk.

Variable	OR (95%CI)		
	Model 1	Model 2	Model 3
Dietary lycopene intake (reference, <P25)			
P25 ~ P50	0.839 (0.728,0.966)	0.847 (0.734,0.978)	0.851 (0.737,0.982)
P50 ~ P75	0.837 (0.724,0.966)	0.823 (0.711,0.953)	0.829 (0.716,0.960)
>P75	0.805 (0.695,0.933)	0.804 (0.692,0.934)	0.807 (0.695,0.938)
Sex (reference, Male)			
Female	1.674 (1.501,1.867)	1.761 (1.568,1.977)	1.796 (1.599,2.018)
Age group (reference, 20–44 years)			
45–64 years	1.312 (1.161,1.483)	1.261 (1.111,1.431)	1.170 (1.028,1.331)
65+ years	0.550 (0.466,0.648)	0.627 (0.526,0.748)	0.558 (0.466,0.668)
Race (reference, Non-Hispanic White)			
Non-Hispanic Black	0.738 (0.642,0.849)	0.753 (0.652,0.869)	0.734 (0.635,0.847)
Mexican American	0.647 (0.548,0.763)	0.819 (0.691,0.970)	0.805 (0.679,0.954)
Other races	0.880 (0.765,1.013)	1.111 (0.961,1.285)	1.095 (0.946,1.267)
Education level (reference, <High school)			
High school or equivalent	0.694 (0.601,0.800)	0.707 (0.611,0.817)	0.719 (0.622,0.832)
>High school	0.567 (0.497,0.647)	0.686 (0.599,0.785)	0.695 (0.607,0.796)
Household income (reference, PIR < 1)			
1 ≤ PIR <4	0.576 (0.513,0.647)	0.626(0.556,0.705)	0.631 (0.560,0.710)
PIR ≥ 4	0.233 (0.192,0.282)	0.288 (0.237,0.350)	0.295 (0.243,0.359)
Marital status (reference, Married/stable union)			
Separate/divorced/widowed	1.846 (1.627,2.094)	1.722 (1.515,1.956)	1.717 (1.511,1.952)
Single	1.375 (1.191,1.588)	1.414 (1.221,1.638)	1.418 (1.224,1.643)
BMI status (reference, Normal or low weight)			
Overweight		1.023 (0.882,1.186)	1.013 (0.873,1.175)
Obese		1.603 (1.404,1.831)	1.496 (1.307,1.712)
Smoking status (reference, Never)			
Former		1.333 (1.153,1.540)	1.310 (1.133,1.515)
Current		2.264 (1.982,2.587)	2.272 (1.989,2.597)
Drinking status (reference, No)			
Yes		1.200 (1.055,1.365)	1.223 (1.074,1.392)
Physical activity (reference, Inactive)			
Active		0.639 (0.573,0.713)	0.656 (0.587,0.732)
HEI-2015 (reference, <P25)			
P25 ~ P50		0.811 (0.707,0.931)	0.808 (0.704,0.928)
P50 ~ P75		0.780 (0.675,0.902)	0.777 (0.672,0.899)
>P75		0.660 (0.560,0.778)	0.659 (0.559,0.776)
Diabetes (reference, No)			
Yes			1.648 (1.428,1.903)

PIR refers to the ratio of household income to poverty; BMI refers to the body mass index; HEI refers to the Healthy Eating Index; P25 refers to the 25th percentile/first quartile; P50 refers to the 50th percentile/median; P75 refers to the 75th percentile/third quartile.

### 3.3 Analysis of restricted cubic spline regression

**Figure 2:**
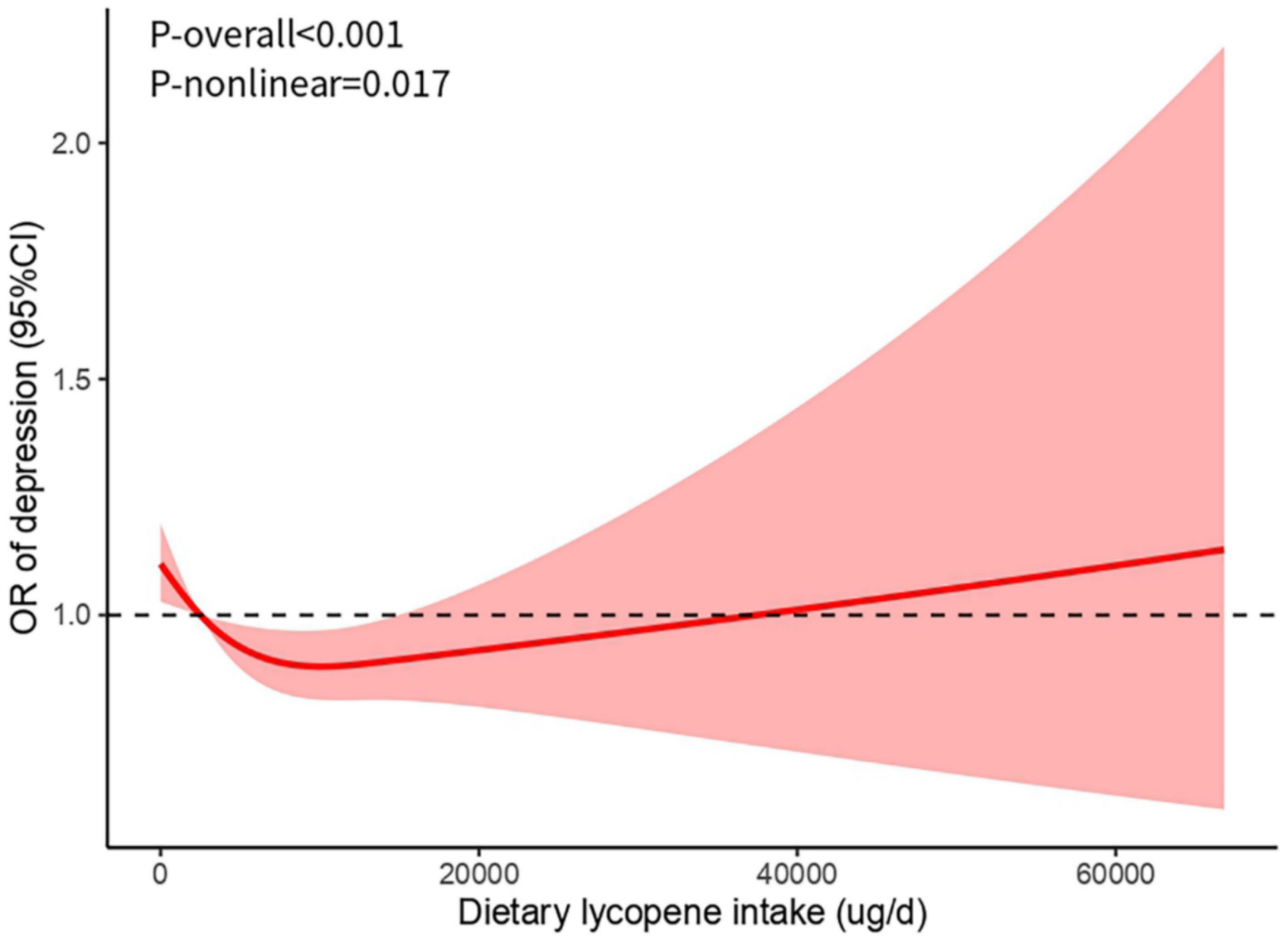
Restricted cubic spline (RCS) model of the association between dietary lycopene intake and depression risk. CI, confidence interval; OR, odds ratio.

The binary logistic regression analysis presented in Table 2 indicated an association between dietary lycopene intake and the risk of depression. To further investigate this relationship, RCS analysis was employed. The RCS results confirmed a significant non-linear trend (*P*-overall <0.001; *P*-non-linear = 0.017) (Figure 2). After adjusting for potential confounders, the analysis demonstrated that within the intake range of 0–10,072 μg/day, higher dietary lycopene intake was associated with a progressively lower risk of depression, consistent with the binary logistic regression findings. However, beyond this threshold, further increases in dietary lycopene intake reverse the beneficial effect of reducing depression risk.

## 4 Discussion

In this study, we investigated the relationship between dietary lycopene intake and depression risk using data from a nationally representative sample of the U.S. population. After comprehensive adjustment for covariates, dietary lycopene intake was consistently associated with a reduced risk of depression across three analytical models. It was also found a threshold point for the protective effect of lycopene against depression.

Existing evidence highlight the multifactorial nature of depression, with diet emerging as a critical modifiable factor (29, 31–34). Several studies have examined the protective role of various carotenoids in depression. A cross-sectional study in American adult population found that levels of five common serum carotenoids were negatively associated with depression risk (35). Similar results have been suggested in studies of older adults, where dietary carotenoid intake was inversely related to depression risk (36–38). A meta-analysis on carotenoid intake and depression risk indicated that higher overall dietary carotenoid intake was associated with a reduced risk of depression (39). However, most of these studies have focused on total carotenoid intake, with few investigations specifically examining the relationship between dietary lycopene intake and depression risk. Lycopene, one of the most potent antioxidants among carotenoids, has garnered significant attention for its potential benefits in neurodegenerative and psychiatric disorders (40–42). A cross-sectional study of individuals aged 70 years and older identified an independent association between a tomato-rich diet and a reduced prevalence of depression, suggesting that such a diet may play a protective role against depression (43). However, it is important to note that older adults are more likely to have multiple diseases, which may affect their mental health, meaning the findings may not be generalizable to the entire population. These findings provide indirect support for the reliability of our results.

The pathophysiology of depression is closely linked to inflammation, with neuroinflammation recognized as a key contributing factor (44–47). Meta-analyses have consistently demonstrated elevated levels of inflammatory markers, such as interleukins and C-reactive protein, in patients with depression, underscoring the role of inflammation in its development (14, 15, 47). Lycopene’s potential antidepressant effects may stem, in part, from its ability to suppress neuroinflammation. Animal studies have shown that lycopene effectively reduces proinflammatory cytokine levels and alleviates neuroinflammatory responses (48, 49). Additionally, lycopene has been reported to improve depressive and anxiety-like behaviors by mitigating neuroinflammation, upregulating neurotrophic factors, and enhancing postsynaptic density protein expression (50).

Depression is also intricately associated with oxidative stress, which arises from an imbalance between reactive oxygen species production and antioxidant defenses. The brain, due to its high oxygen consumption, lipid-rich composition, and relatively weak antioxidant defenses, is particularly vulnerable to oxidative stress, resulting in structural and functional neuronal damage (12). Lycopene has been shown to attenuate oxidative stress and endoplasmic reticulum stress by reducing oxidative markers and inhibiting activation of the protein kinase-like endoplasmic reticulum kinase signaling pathway (51).

In summary, lycopene exerts its beneficial effects on depression through its dual roles in modulating inflammation and oxidative stress, supporting its potential as a dietary strategy for the prevention and management of depression. Notably, the results also revealed an inversion phenomenon in the correlation observed, occurring upon surpassing a particular threshold. This inversion may be attributed to the oversaturation of antioxidant activity. Lycopene, a highly efficacious antioxidant, primarily functions to alleviate oxidative stress by neutralizing free radicals (52). Nevertheless, as the quantity of dietary lycopene intake escalates, the antioxidant system attains a state of saturation, rendering any additional lycopene ineffective in further augmenting antioxidant effects, and consequently, diminishing its overall benefits. This discovery underscores the importance of regulating dietary lycopene intake within a prudent range to maximize its health-promoting benefits.

This study has several notable strengths. First, it utilizes a large, nationally representative sample from NHANES 2007–2016, making it the largest study to explore the relationship between dietary lycopene intake and depression risk. It fills a crucial gap in the epidemiological evidence regarding the association between the two. Second, by combining results from binary logistic regression and RCS models, we have provided evidence of a negative association between them. Third, our study reveals a potential non-linear relationship between dietary lycopene intake and depression risk, identifying a threshold point for the protective effects of lycopene (10,072 μg/day). In conclusion, the findings not only confirm the potential impact of lycopene on depression risk but also offer new perspectives on the role of dietary interventions in psychological health management.

It is important to acknowledge several limitations in this study. First, the cross-sectional design of the study limits the ability to establish causal relationship. Future research should consider designing a cohort study to examine how baseline levels of dietary lycopene intake affect depression risk, which could help clarify the causal relationship. Second, reliance on self-reported 24-h dietary recall data and the PHQ-9 questionnaire introduces the potential for recall bias and reporting bias, which may affect the accuracy of dietary and depression assessments. Furthermore, although this study has accounted for multiple factors in the analysis, there are still unmeasured variables, such as genetic susceptibility, medication use, and other dietary components, which may influence the interpretation of the results. Therefore, the inability to control for these potential confounding factors represents another limitation of this study.

In conclusion, the findings of this study highlight lycopene as a promising dietary intervention worthy of integration into public health strategies. The promotion of increased lycopene consumption may serve as an innovative approach to depression prevention and management, thereby contributing to the enhancement of population mental health outcomes. To optimize the preventive potential of lycopene against depression, achieving an optimal intake level is essential. Based on the analytical results of this study, a daily dietary lycopene intake of approximately 10 mg is recommended for adults. Consuming lycopene with foods rich in healthy fats is advised, as this enhances its absorption and bioavailability. A diversified diet, including an increased intake of tomato-based products, represents an effective strategy for meeting daily lycopene requirements. This study provides novel evidence supporting the role of lycopene in mental health applications. Further research and public health efforts should focus on raising awareness of lycopene’s benefits and encouraging dietary patterns that support mental health.

## 5 Conclusion

Despite its limitations, our study first reports an independent association between dietary lycopene intake and the risk of depression, providing a specific intake range (0–10,072 μg/day). This finding addresses the gap in epidemiological evidence linking them and may offer new dietary intervention strategies to reduce the risk of depression. These results provide theoretical support for promoting dietary patterns beneficial to mental health. However, further large-scale prospective studies are required to explore the role of lycopene in depression.

## Data Availability

The original contributions presented in the study are included in the article/supplementary material, further inquiries can be directed to the corresponding author.
